# Efficacy of injectable platelet-rich fibrin (i-PRF) as a novel vehicle for local drug delivery in non-surgical periodontal pocket therapy: A randomized controlled clinical trial

**DOI:** 10.34172/japid.2024.021

**Published:** 2024-09-11

**Authors:** Murugan Thamaraiselvan, Nadathur Doraisamy Jayakumar

**Affiliations:** ^1^Department of Periodontics, Saveetha Dental College & Hospitals, Saveetha Institute of Medical and Technical Sciences, Chennai, Tamilnadu, India

**Keywords:** Drug carriers, Platelet-rich fibrin, Periodontal disease

## Abstract

**Background.:**

The vehicle in a local drug delivery (LDD) system plays a vital role in delivering the active drug component at the diseased site. Liquid/injectable platelet-rich fibrin (i-PRF), an autologous fibrin matrix, might be used as a vehicle to enmesh drugs and deliver locally at the periodontally diseased sites. This study evaluated the efficacy of the drug (ciprofloxacin [Cip])-loaded i-PRF as a LDD system adjunct to subgingival debridement in subjects with periodontal pockets.

**Methods.:**

In a parallel design study, 79 periodontally diseased pocket sites were randomized to 3 groups: group 1 (n=25), scaling and root planing (SRP)+i-PRF+Cip; group 2 (n=25), SRP+i-PRF; group 3 (n=25), SRP without any adjunctive intervention. Clinical parameters (probing depth [PD], clinical attachment level [CAL], gingival index [GI], plaque index [PI]) and microbial quantification (relative quantification of levels of *Aggregatibacter actinomycetemcomitans*) were assessed from baseline to 6th and 12th weeks of follow-up.

**Results.:**

All the treatment groups showed significant improvements in the clinical and microbial parameters assessed. Group 1 showed significantly higher PD and GI reduction with CAL gain and decreased in relative levels of *A. actinomycetemcomitans* in the 12th week, followed by group 2 compared to group 3.

**Conclusion.:**

Thus, within the limits of this study, it can be concluded that i-PRF could be considered a potential LDD vehicle for the delivery of ciprofloxacin in periodontal pocket therapy.

## Introduction

 Periodontitis is an inflammatory disease of the supporting structures of the teeth with a microbial origin and modified by multiple host and environmental factors.^1,2^ Polymicrobial biofilm is a prerequisite for the initiation of the disease, resulting in a host‒microbial interaction, leading to the destruction of host tissues, including alveolar bone, cementum, and periodontal ligament.^3^ Although conventional mechanical periodontal therapy (scaling and root planing [SRP])^4^ targets eliminating these microbial biofilms with the reversal of the inflammatory process, clinical scenarios like deep pockets around inaccessible/difficult-to-access areas like furcation might pose limitations in the complete removal of the plaque biofilm, resulting in residual microbiota within the pocket, favoring further progression of periodontal disease.^5,6^ Additionally, the inherent nature of certain pathogens like *Porphyromonas gingivalis* to infiltrate the connective tissue of the periodontal pockets can potentially repopulate the debrided pocket, resulting in relapse.^7^ Furthermore, host factors like excessive, uncontrolled, or defective immune responses seen in diabetics and smokers might result in continuous tissue destruction with impaired tissue repair.^8,9^ All these situations demand an additional intervention to the conventional mechanical periodontal therapy, like systemic agents including antibiotics or host-modulating drugs that target the residual periodontal pathogens or the host immune and inflammatory responses, respectively, together constituiting adjunct periodontal therapies.^10^ However, apart from their promising improved clinical benefits compared to conventional mechanical therapy,^11,12^ they result in more frequent adverse events like antibiotic resistance and other systemic side effects that have questioned the risk-benefit ratio.^13,14^

 In periodontal therapy, local drug delivery (LDD) has been in practice for the past three decades in treating localized pockets, showing improved clinical and microbiological parameters comparable to adjunctive systemic antibiotic therapy.^9,10^ Additionally, it has the benefits of a low dose of drug use sufficient to attain the required minimal inhibitory concentrations (MIC) in periodontal tissue, absence of systemic effects, sustained release of drugs, absence of resistance formation, etc.^14^ Although the current LDD systems like fibers, chips, and gels have overcome the shortcomings of systemic therapy, they still pose some challenges like synthetic or exogenous origin, time consumed for the placement (fibers), a local inflammatory reaction to the degraded products, discomfort to the patient, chances of displacement/dislodgement from the site, need to be removed after the therapy, high cost and some reports (unclear data) about transient antimicrobial resistance.^15-17^ Newer systems like in-situ gel formulations (hydrogels, polysaccharides, and polymers) with unique properties of sol-to-gel conversion, when influenced by biological stimuli (pH, temperature, and ion exchange) are reported to be more biocompatible and easy to handle.^17,18^ However, challenges like the complexity of the system, responsiveness to bio-stimulus, reproducibility in terms of performance, lack of tissue integration, and high cost prevent its subsequent translation to clinical use.^19^ Apart from these, the non-degradability of most synthetic in situ gel formulations also poses an additional challenge to the above list.^19^ Hence, it is recommended to switch to in situ gel formulations of natural polymer origin that are non-toxic and biodegradable. Thus, the search for an ideal vehicle in periodontal therapy is still continuing.

 In this context, platelet-rich fibrin (PRF), a second-generation platelet concentrate,^20,21^ is widely used in oral, dental, and periodontal regenerative applications. It has a fibrin matrix framework that enmeshes platelets, leukocytes, growth factors (GFs), and other cytokines.^22^ A modification of the centrifugation protocol results in a liquid form of PRF (liquid PRF) that slowly polymerizes to become a fibrin clot (gel) after approximately 3 minutes, which can be injected into tissues.^23^ In both forms of PRF, the fibrin matrix plays a crucial role by mimicking a 3D scaffold loaded with growth factors, which gradually degrades, resulting in sustained release of the content into the regeneration area.^24-29^ Liquid/i-PRF, apart from being syringeable and injectable, is also an autologous product that shows good tissue integration and can facilitate the localization of the PRF clot with the required site. Considering the above benefits (sol-to-gel transition, injectable, autologous, bioadhesive [tissue integration]) and the 3D architecture of the iPRF, we thought that it could be used as a vehicle to enmesh drugs and deliver locally at the periodontally diseased sites. Our recent in vitro study using i-PRF as a vehicle for local delivery of three drugs (ciprofloxacin, curcumin, and tannic acid) showed a sustained release pattern of all three drugs with only 59%, 64%, and 20% of the loaded drug released at the end of the observation period (14 days). These study results lead us to continue this human clinical trial. Thus, this study evaluated the efficacy of the drug (ciprofloxacin [Cip])-loaded i-PRF as a LDD system adjunct to subgingival debridement in subjects with periodontal pockets.

## Methods

**Figure 1:**
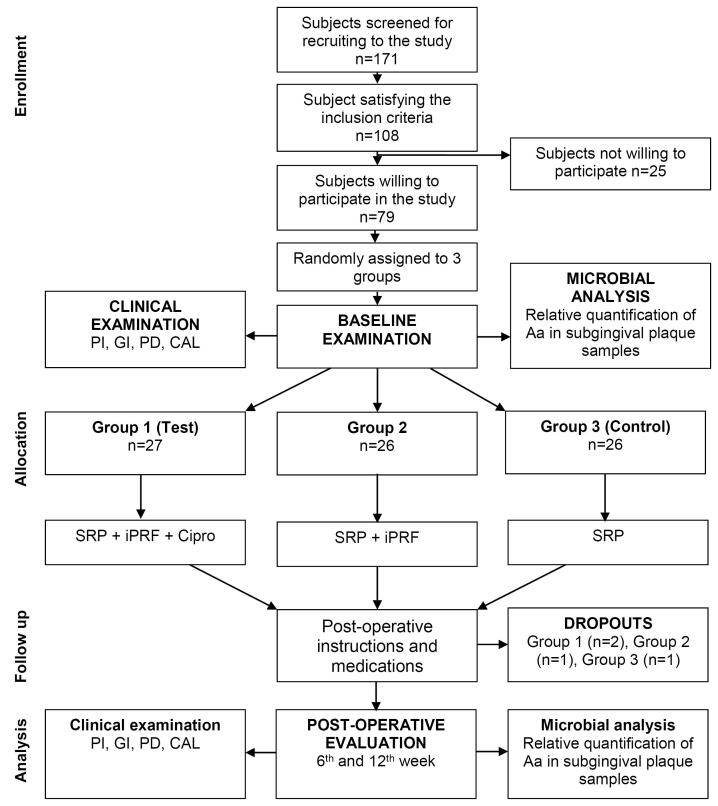
Flowchart showing the study design and protocol (CONSORT Diagram)

 The present single-centered, randomized, controlled parallel-design study was carried out in the Department of Periodontics at a university dental hospital setting adhering to CONSORT guidelines (Figure 1). After the study design was approved by the institutional Ethics Committee and institutional Review Board (1HEC Ref No: IHEC/SDC/PERIO-1802/22/573), it was registered in the clinical trial registry (http://ctri.nic.in) (CTRI/2023/01/048659 [Registered on: 02/01/2023]). The study protocol also follows Helsinki’s declaration for human trials as revised in 2008.

 The sample size for the clinical trial was calculated using the formula reported by Chow et al.^30^ The estimation indicated that at least a sample size of 12 is required to gain 80% power and show a difference in mean probing depth (PD) between 3 groups at the end of 12 weeks of evaluation. A final sample of n = 25 in each group was decided to compensate for the dropouts during the study.

 The study population was selected from the subjects visiting the outpatient section of the Department of Periodontics at Saveetha Dental College and Hospitals between January 2023 and March 2023 based on the following inclusion and exclusion criteria.

###  Inclusion criteria

1. The age group of 30‒60 years
2. Subjects diagnosed with Stage II Grade B periodontitis based on the 2018 classification31
3. Subjects with at least 20 teeth at the time of initial examination
4. Subjects with periodontal condition showing PD of ≥ 5 mm in at least two sites
5. Subjects with periodontal conditions showing clinical attachment loss (CAL) of ≥ 2 mm in at least two sites

###  Exclusion criteria

1. Pregnant and lactating women
2. Use of immunosuppressive medications, consumption of antibiotics, and any antioxidants and anti‐inflammatory agents in the last three months
3. A history of periodontal therapy in the preceding one year
4. Subjects with hemoglobin levels < 11 mg/dL
5. Subject participating in any other clinical trials

A total of 108 subjects meeting the study criteria were briefed about the study protocol and asked for written consent. Of these, 79 subjects gave written consent to participate in the study, each with one experimental site contributing to 79 sites.

###  Baseline examination

 For all the enrolled participants, baseline plaque samples were collected according to the standard protocol at the experimental site, as follows, by a single blinded examiner. After supragingival plaque removal using a curette, adequate isolation was achieved around the experimental sites using cotton rolls.

- Plaque samples were collected from the subgingival environment using #25 sterile paper points placed into the experimental pocket site to reach the bottom and left in place for 15 seconds. Then, the paper point was transferred to a vial containing 1 mL of viability medium Gothenburg anaerobic (VMGA) transport medium and sent to the laboratory under anaerobic conditions immediately. After vortexing for 60 seconds, the samples were used for the polymerase chain reaction (PCR) analysis.

This was followed by clinical data collection using a Williams periodontal probe as follows:

- Plaque index (PI) based on Silness and Löe (1964)
- Gingival index (GI) based on Silness and Löe (1963)
- PD measured from the marginal gingiva to the base of the pocket
- Clinical attachment level (CAL) measured from the cementoenamel junction to the base of the pocket

All the participants underwent phase 1 periodontal therapy, Including complete ultrasonic scaling with root planing at localized periodontal pocket sites with Gracey curettes by a single operator. Following this, periodontal pockets were irrigated with copious saline to flush the disrupted biofilm and calculus out of the pocket environment.

 These subjects were then randomly assigned to one of the three groups using computer-generated numbers using the SAS^®^. The allocation concealment for the groups was done using sequentially numbered, sealed, opaque envelopes. All the randomization and allocation concealment were done by a separate examiner (JK) other than the principal investigator.

- Group 1 – Drug-loaded injectable platelet-rich fibrin (i-PRF): After SRP, this group was treated with the delivery of ciprofloxacin-loaded i-PRF into the periodontal pocket and gingival tissue adjacent to the pocket wall. The i-PRF was applied only once and was not repeated during the study period.
- Group 2 – Drug-free i-PRF: After SRP, this group was treated with the delivery of drug-free i-PRF into the periodontal pocket and gingival tissue adjacent to the pocket. The i-PRF was applied only once and was not repeated during the study period.
- Group 3 – SRP: This group was treated only with SRP without any adjunct therapy.

Once the allocation was done, for the participants in group 3, this was the only therapy rendered. In contrast, for the participants in groups 1 and 2, i-PRF with and without ciprofloxacin was applied as an adjunct to SRP local delivery, respectively. The i-PRF and i-PRF loaded with ciprofloxacin were prepared as follows.

###  Collection of i-PRF

 The iPRF was prepared by the same operator according to the protocol developed by Miron and Choukron in 2017.^32^ It involves collecting 10 mL of intravenous blood from the participant using venipuncture of antecubital vein under sterile conditions. The collected blood is transferred to a plain sterile test tube without anticoagulant and immediately subjected to centrifugation at 70-g force at 700 rpm for 3 minutes. After centrifugation, the blood separates into two parts: the bottom layer consists of a red blood cell compartment, and the top layer is PRF plasma, which is still in liquid consistency. The top PRF layer is aspirated into a 2-mL syringe and maintained in liquid consistency for about 3‒5 minutes until it clots by slow polymerization of fibrin formation.

###  Preparation of the drug solution

 The concentration of the ciprofloxacin drug to be loaded in iPRF was decided based on our team’s earlier in vitro cytocompatibility and drug release kinetic study (unpublished data). According to the data obtained, 1 mg/mL of the drug concentration was found to be biocompatible with maximum efficacy and showed a sustained release of 59% of loaded drug at the end of 14-day observation. Analytical grade ciprofloxacin hydrochloride was obtained from Sigma-Aldrich Corp. (Milwaukee, Wis., USA). One milligram of the drug was weighed and mixed with 100 µL of deionized water and shaken for 30 seconds to make the drug completely soluble, which was done just before the blood collection from the participants.

###  Preparation of the drug-loaded i-PRF

**Figure 2:**
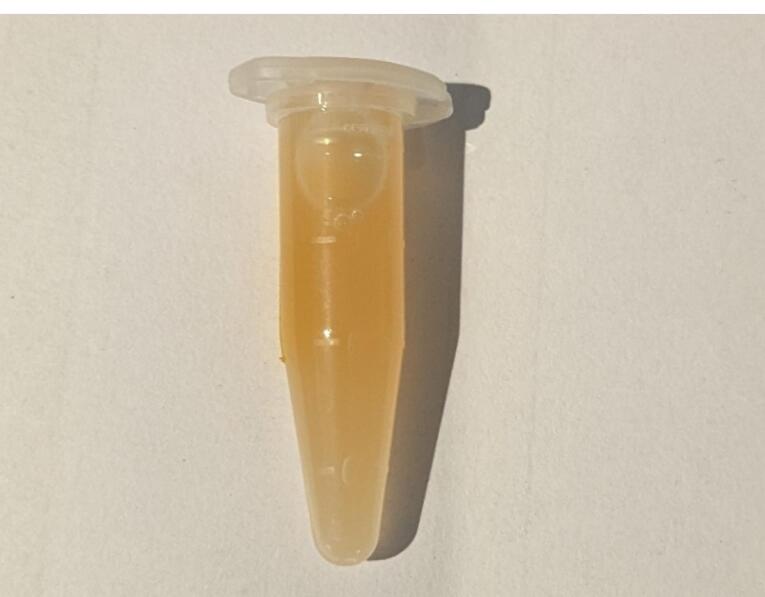
i-PRF mixed with ciprofloxacin drug in liquid consistency

 Then, 900 of the obtained i-PRF was dispensed in a vial containing a 1-mg/100 µL solution of ciprofloxacin and shaken gently for 10 seconds to obtain a homogenous mix with a final concentration of 1 mg/mL (Figure 2).

###  Local delivery of ciprofloxacin-loaded i-PRF

**Figure 3:**
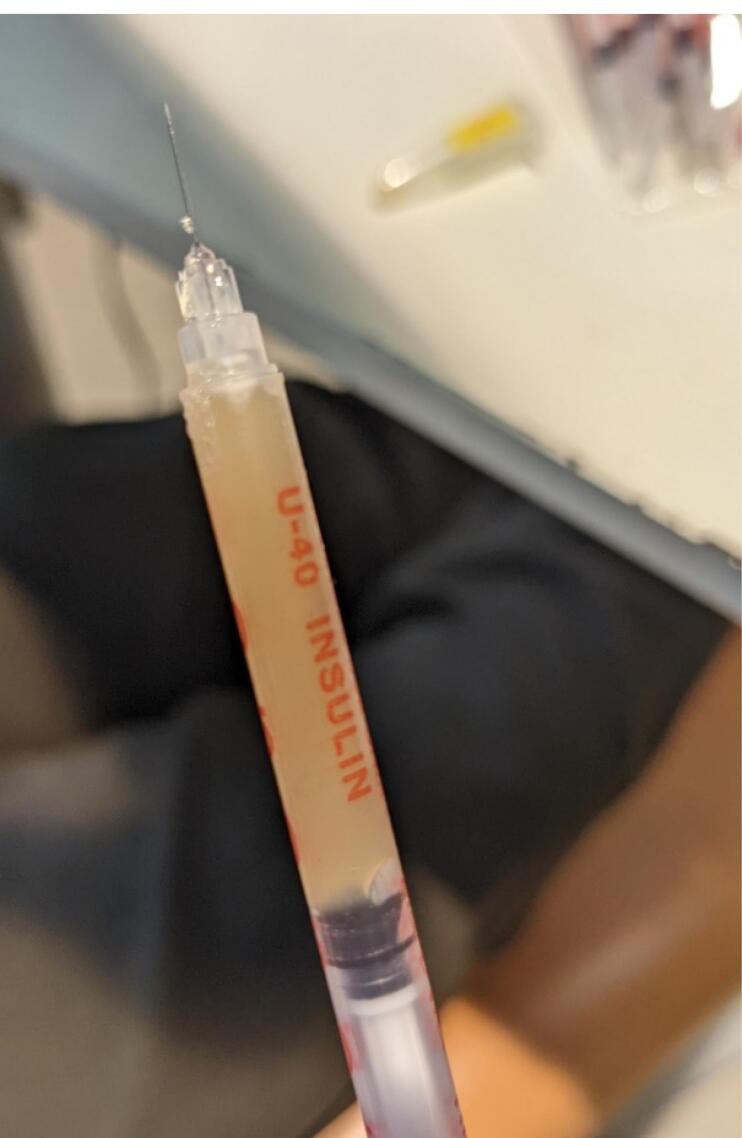
i-PRF mixed with ciprofloxacin loaded in an insulin syringe

**Figure 4:**
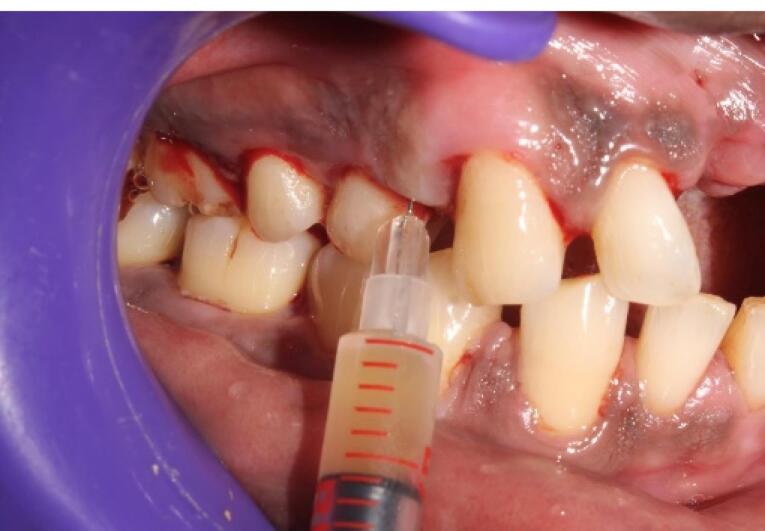
i-PRF mixed with ciprofloxacin injected into the periodontal pocket (group 1)

This mixture was further immediately loaded in a 1-mL insulin syringe (Figure 3) and injected into the periodontal pocket until it filled and overflowed followed by injecting into the tissues adjacent to the periodontal pocket before it became a gel in the participants of group 1 (Figure 4). In group 2 participants, plain iPRF was delivered at the experimental sites as explained above and allowed to gel. Postoperative instructions were given to all the study participants. There was no prescription for mouthwash or medications for any of the subjects, and they were asked to report after 6 and 12 weeks for follow-up.

###  Follow-up examinations

 All the clinical measurements and plaque sample collection procedures were performed by the same calibrated examiner (SS) with an intra-observer (first and second readings) reliability expressed as a weighted Kappa coefficient with a 95% confidence interval. The operator was blinded throughout the study. The plaque sample collection and clinical examination (PD, CAL, GI, and PI) were performed at the 6th- and 12th-week follow-ups.

###  Microbial analysis

 From the plaque samples collected from the subgingival area, relative quantification of the level of *Aggregatibacter actinomycetemcomitans* in the total level of bacterial cells was assessed using TaqMan real-time polymerase chain reaction (RT-PCR). The DNA extraction and isolation were performed using a standardized method described in the laboratory manual for molecular cloning from the plaque biofilm samples.^33^ The *A. actinomycetemcomitans* (ATCC 29523) bacterial DNA identification was performed using the bacterial DNA template and bacteria-specific primers and probes that were processed as recommended by the manufacturer with the following amplification protocols (50 °C for 2 minutes, 95 °C for 10 minutes, and then 60 cycles of 15 seconds at 95 °C and 1 minute at 58 °C). The sequence and PCR procedures have been previously described in detail by Kato et al.^34^ For the relative quantification, the copy numbers of pathogenic bacterial genes were standardized to the copy number of the 16S rRNA genes by using the simplified comparative threshold cycle (∆Ct) method reported by Yoshida et al.^35^

###  Primary and secondary outcomes

 The primary outcomes are the changes in clinical parameters, such as a reduction in PD, which was evaluated in the 12th week. The secondary outcomes are changes in PI, GI, gain in CAL, and relative quantity of *A. actinomycetemcomitans.*

###  Statistical analysis

 The results of the clinical parameters are expressed as mean ± standard deviation. All the parameters were compared within groups at different time intervals using one-way ANOVA, while inter-group comparison was made using ANOVA followed by post hoc analysis by the Bonferroni test. The microbial analysis reported the relative proportion of *A. actinomycetemcomitans* bacterial cell counts at different time intervals as mean counts (log10) ± SD. Comparisons between groups at different time intervals were made using one-way ANOVA, while comparisons between groups were made using ANOVA.

## Results

**Figure 5:**
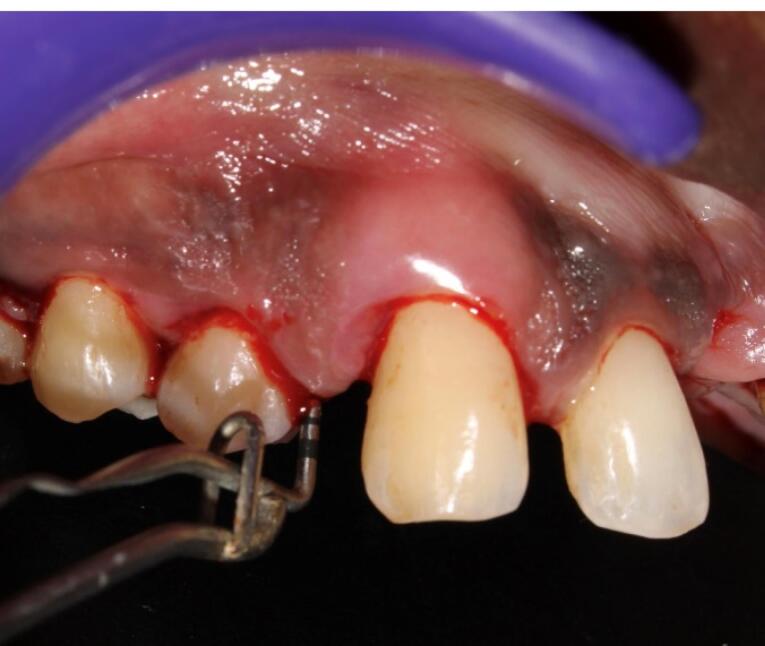
Preoperative picture (Baseline) showing PD in one of the experimental sites (#5) (group 1)

**Figure 6:**
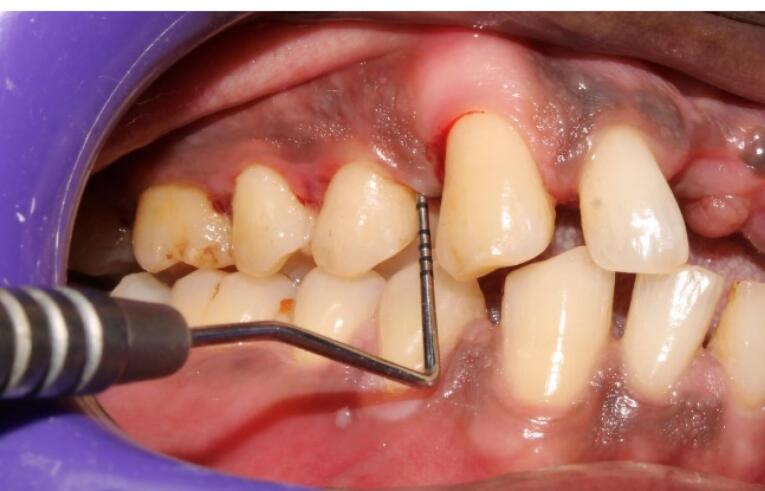
Postoperative picture (12th week) showing reduced PD in the experimental site (#5) shown in Figure 5 (group 1)

 All the recruited subjects underwent the intervention (group 1 [n = 27], group 2 [n = 26], group 3 [n = 26]). During the follow-up, there were two dropouts from group 1 and one from each of the groups 2 and 3, resulting in 25 final samples in each group to be considered for final statistical analysis. No adverse reactions were observed during the study period for any of the interventions, and none of the participants reported any discomfort with the treatment protocol (Figures 5 and 6). The results are shown as mean ± standard deviation (SD) for clinical parameters and mean counts (log10) ± SD for microbiological parameters in Table 1 to Table 5.

**Table 1:** Comparison of probing depths (PD) within and between groups

**PD (mm)**	**Group 1 (n=25)**	**Group 2 (n=25)**	**Group 3 (n=25)**	***P*** **value**
Baseline	5.66 ± 0.63	5.45 ± 0.65	5.83 ± 0.76	0.893
6th week	3.91 ± 0.58^‡§^	4.41 ± 0.65^§^	4.87 ± 0.79^§^	0.413
12th week	3.70 ± 0.46^‡§^	4.33 ± 0.56^†§^	4.79 ± 0.72^§^	0.002^‖^

PD: probing depth; group 1: drug-loaded i-PRF (injectable platelet-rich fibrin); group 2: drug-free i-PRF; group 3: SRP (scaling and root planing). ^†^Statistically significant (*P* < 0.05) compared to group 3. ^‡^Statistically highly significant (*P* < 0.01) compared to group 3. ^§^Statistically significant (*P* < 0.05) compared to baseline. ^‖^Statistically significant (*P* < 0.05) when compared between groups 1 and 2.

**Table 2:** Comparison of clinical attachment level (CAL) within and between groups

**CAL (mm)**	**Group 1 (n=25)**	**Group 2 (n=25)**	**Group 3 (n=25)**	***P*** **value**
Baseline	4.54 ± 0.58	4.37 ± 0.71	4.66 ± 0.70	1.000
6th week	2.33 ± 0.56^‡§^	2.91 ± 0.77^‡§^	3.91 ± 0.65 ^§^	0.117
12th week	2.33 ± 0.56^‡§^	2.91 ± 0.77^†§^	4.00 ± 0.65^§^	0.011^‖^

CAL: clinical attachment level; group 1: drug-loaded i-PRF (injectable platelet-rich fibrin); group 2: drug-free i-PRF; group 3: SRP (scaling and root planing). ^†^Statistically significant (*P* < 0.05) compared to group 3. ^‡^Statistically highly significant (*P* < 0.01) compared to group 3. ^§^Statistically significant (*P* < 0.05) compared to baseline. ^‖^Statistically significant (*P* < 0.05) when compared between groups 1 and 2.

**Table 3:** Comparison of gingival index (GI) within and between groups

**GI (%)**	**Group 1 (n=25)**	**Group 2 (n=25)**	**Group 3 (n=25)**	***P*** **value**
Baseline	1.66 ± 0.48	1.87 ± 0.33	1.66 ± 0.48	0.314
6th week	0.85 ± 0.15^‡§^	0.85 ± 0.16^‡§^	1.15 ± 0.12^§^	1.000
12th week	0.91 ± 0.22^‡§^	0.88 ± 0.14^‡§^	1.15 ± 0.12^§^	1.000

GI: gingival index; group 1: drug-loaded i-PRF (injectable platelet-rich fibrin); group 2: drug-free i-PRF; group 3: SRP (scaling and root planing). ^†^Statistically significant (*P* < 0.05) compared to group 3. ^‡^Statistically highly significant (*P* < 0.01) compared to group 3. ^§^Statistically significant (*P* < 0.05) compared to baseline. ^‖^Statistically significant (*P* < 0.05) when compared between groups 1 and 2.

**Table 4:** Comparison of plaque index (PI) within and between groups

**PI (%)**	**Group 1 (n=25)**	**Group 2 (n=25)**	**Group 3 (n=25)**	***P*** **value**
Baseline	1.45 ± 0.50	1.57 ± 0.49	1.50 ± 0.51	1.000
6th week	0.74 ± 0.19^§^	0.65 ± 0.16^§^	0.64 ± 0.10^§^	0.144
12th week	0.68 ± 0.09^§^	0.65 ± 0.08^†§^	0.69 ± 0.10^§^	1.000

PI: plaque index;group 1: drug-loaded iPRF (injectable platelet-rich fibrin); group 2: drug-free i-PRF; group 3: SRP (scaling and root planing); BL, baseline. ^†^Statistically significant (*P* < 0.05) compared to group 3. ^‡^Statistically highly significant (*P* < 0.01) compared to group 3. ^§^Statistically significant (*P* < 0.05) compared to baseline. ^‖^Statistically significant (*P* < 0.05) when compared between groups 1 and 2.

**Table 5:** Comparison of *Aggregatibacter actinomycetemcomitans* levels within and between groups

***A. actinomycetemcomitans* (Mean counts (log10)±SD)**	**Group 1 (n=25)**	**Group 2 (n=25)**	**Group 3 (n=25)**	***P*** **value**
Baseline	0.97 ± 0.21	1.04 ± 0.37	1.02 ± 0.31	1.000
6th week	0.14 ± 0.10^†§^	0.27 ± 0.11^†§^	1.27 ± 0.52	0.03 ^‖^
12th week	0.19 ± 0.17^†§^	0.35 ± 0.12^†§^	1.32 ± 0.41	0.03 ^‖^

PI: plaque index; group 1: drug-loaded iPRF (injectable platelet-rich fibrin); group 2: drug-free i-PRF; group 3: SRP (scaling and root planing). ^†^Statistically significant (*P* < 0.05) compared to group 3. ^‡^Statistically highly significant (*P* < 0.01) compared to group 3. ^§^Statistically significant (*P* < 0.05) compared to baseline. ^‖^Statistically significant (*P* < 0.05) when compared between groups 1 and 2

## Discussion

 Adjunctive use of LDD to SRP in periodontal pocket therapy has been documented thoroughly in the literature, with most of the evidence supporting its additional beneficial effect.^36,37^ The current research in LDD is more inclined toward developing an ideal vehicle that facilitates sustained release with high biocompatibility with the host tissue.^38^ The rationale for using iPRF as an LDD vehicle for pocket therapy in the present study stemmed from its mesh-like fibrin architecture that has shown a sustained release of entrapped growth factors at the periodontal wound sites,^39,40^ with extensive biological activities and advanced safety margin. To our knowledge, this is the first study in the literature to evaluate iPRF as an LDD system in periodontal pocket therapy.

 Our study involved a parallel design protocol appropriate for LDD investigations to eliminate the crossover effect.^41^ Also, our sample distribution showed no significant difference in baseline clinical parameters between the groups, suggesting the elimination of selection bias. The clinical and microbiological outcomes for the intervention were assessed for three months, which was reported to be sufficient to investigate the effect of the LDD.^42,43^

 All the subjects in our study showed good oral hygiene after being included in the study protocol, which was evident in the significant reduction in the PI score concurrent with earlier studies.^44^ This might be due to the oral hygiene instruction and reinforcement given after scaling protocol and the recruitment of only the subjects who showed satisfactory oral hygiene.

 Similarly, there was a significant improvement in GI irrespective of the intervention made in all the treatment groups.^45^ The resolution of gingival inflammation should have been mediated by the elimination of subgingival plaque biofilm by SRP and improved oral hygiene by the patient in all the treatment groups.^45^ However, incorporating i-PRF into SRP in groups 1 and 2 resulted in further improvements in GI, which concurs well with earlier reports.^46^ This may be attributed to the anti-inflammatory effect of the white blood cells and the cytokine released from it.^46^ The presence of ciprofloxacin in group 1 did not bring about any further resolution of gingival inflammation compared to group 2, suggesting that most of the gingival inflammatory resolution was mediated by the anti-inflammatory effect of i-PRF rather than ciprofloxacin.

 All our interventions resulted in a significant PD reduction, with group 1 showing the maximum reduction, followed by group 2 compared to group 3 at the end of the 12th week. This is concurrent with earlier reports where SRP resulted in a significant reduction in PD, which was mainly attributed to the resolution of periodontal inflammation after the elimination of the plaque biofilm.^45^ Furthermore, the adjunctive use of i-PRF showed a significant PD reduction, which is supported by previous studies.^46^ This could be due to the delivery of growth factors and other anti-inflammatory cytokines from the PRF matrix to the periodontal pocket that may reduce inflammation and enhance the regenerative potential at the pocket environment, which could have resulted in PD reduction. The highest PD reduction in group I could be due to the antibacterial effect of ciprofloxacin delivered by the i-PRF vehicle. Since no previous studies are available, a direct comparison of our study outcome was not possible. Nevertheless, our results are consistent with earlier reports on other LDD systems showing a significant PD reduction when LDD was used.^47^ Furthermore, the ciprofloxacin-loaded group (group 1) outperformed the plain i-PRF LDD group (group 2), indicating a possible role of the drug released from the i-PRF vehicle apart from the i-PRF itself.

 Evaluation of CAL showed significant attachment gain in all the treatment groups at the 12th week of observation, consistent with earlier reports^46,48^ where SRP facilitates attachment gain through the formation of long junctional epithelium.^49^ Among all the groups, the ciprofloxacin-loaded i-PRF group (group 1) showed a significant maximum attachment gain compared to other groups. A possible explanation for this is the potential of fibrin in i-PRF to adhere to the root surface, which could have facilitated a new attachment. However, this should be confirmed with further histologic investigation. Nevertheless, the role of ciprofloxacin, resulting in maximum attachment gain compared to the plain iPRF group, needs to be evaluated.

*Aggregatibacter actinomycetemcomitans* is one of the primary periodontal pathogens involved in the pathogenesis of periodontal destruction and pocket formation. Hence, measuring it quantitatively *(A. actinomycetemcomitans*) could be one of the valuable methods for monitoring periodontal disease status and assessment for treatment outcomes. Also, ciprofloxacin, being a broad-spectrum agent and reported to be active against all strains of *A. actinomycetemcomitans*, could be a validation for the microbial analysis. All our experimental groups resulted in a decrease in the relative levels of* A. actinomycetemcomitans*, with group 1 showing the maximum changes, followed by groups 2 and 3. As mentioned earlier, the lack of studies limits the direct comparison of our results. However, this is similar to other reports on LDD with fluoroquinolones, significantly reducing *A. actinomycetemcomitans* levels.^47^ Between groups 1 and 2, the former resulted in a significant reduction of *A. actinomycetemcomitans* than the latter. This tendency ensures the possible role of ciprofloxacin in microbial reduction and, in turn, results in improvements in clinical parameters. A possible explanation for the improved clinical and microbiological parameters for three months (12 weeks) could be by the natural biodegradation of the fibrin network of i-PRF fibrin matrix through enzymatic or hydrolytic tissue reaction that could result in gradual and sustained release of ciprofloxacin in the periodontally infected sites thus ensuring the extended availability of the drug.

 Apart from the clinical efficacy observed, there are many advantages of using an i-PRF vehicle, which differs from conventional vehicles (synthetic/natural or exogenous) since it is autologous, easy to handle (can be prepared chairside, with no addition of any chemicals, syringeability), and can be injected directly into the periodontal tissues and pocket. Its slow polymerization to gel allows incorporation of the drug and polymerization within the pocket, resulting in the gel’s better adaptation to periodontal pocket dimensions. Additionally, the adherence and integration of i-PRF to the host tissue, like the root surface and gingival tissue, minimizes the dislodgement of the vehicle, thus ensuring sustained drug delivery and, finally, its economic benefits.

 Future research to confirm the sustained release by evaluating the availability of loaded drugs in the oral environment is mandatory to add strength to the current evidence. Also, evaluation of the influence of loaded drugs on the biological nature of the i-PRF vehicle has to be ascertained. Other limitations are the shorter length of follow-up and the absence of a positive control group.

 From the above observations, the use of ciprofloxacin-loaded i-PRF as an LDD system resulted in a significant reduction of *A. actinomycetemcomitans,* which in turn contributed to clinical improvements in terms of PD, GI reduction, and CAL gain. Thus, within the limits of this study, it can be concluded that i-PRF could be considered a potential LDD vehicle for the delivery of ciprofloxacin in periodontal pocket therapy.

## Acknowledgments

 The authors would like to acknowledge the postgraduate resident, Deepika, for her support in scheduling the patients for treatment and follow-up appointments.

## Competing Interests

 The authors declare that they have no competing interests.

## Consent for Publication

 Written consent was obtained from all the study subjects for the use of pictures and data during publication.

## Data Availability Statement

 Individual deidentified participant data collected (Study protocol, informed consent form) during the study will be shared after deidentification; the data will become available immediately after publication for three months for researchers who provide a methodologically sound proposal; data will be shared on request by mail.

## Ethical Approval

 The study design was approved by the institutional ethical committee and institutional review board (1HEC Ref No: IHEC/SDC/PERIO-1802/22/573) of Saveetha Dental College and Hospitals, Saveetha Institute of Medical and Technical Sciences.
